# Implementation of a dual-phase grating interferometer for multi-scale characterization of building materials by tunable dark-field imaging

**DOI:** 10.1038/s41598-023-50424-6

**Published:** 2024-01-03

**Authors:** Caori Organista, Ruizhi Tang, Zhitian Shi, Konstantins Jefimovs, Daniel Josell, Lucia Romano, Simon Spindler, Pierre Kibleur, Benjamin Blykers, Marco Stampanoni, Matthieu N. Boone

**Affiliations:** 1 Radiation Physics Research group, Department Physics and Astronomy, Ghent University, 9000 Ghent, Belgium; 2 Centre for X-ray Tomography, Ghent University, 9000 Ghent, Belgium; 3 UGent‑Woodlab, Department of Environment, Faculty of Bioscience Engineering, Ghent University, 9000 Ghent, Belgium; 4 Pore-Scale Processes in Geomaterials Research Group (PProGRess), Department of Geology, Ghent University, 9000 Ghent, Belgium; 5 Institute for Biomedical Engineering, ETH Zurich, 8092 Zurich, Switzerland; 6 Swiss Light Source, Paul Scherrer Institute, Villigen, 5232 Switzerland; 7 Materials Science and Engineering Division, National Institute of Standards and Technology, Gaithersburg, MD USA

**Keywords:** Engineering, Materials science, Optics and photonics

## Abstract

The multi-scale characterization of building materials is necessary to understand complex mechanical processes, with the goal of developing new more sustainable materials. To that end, imaging methods are often used in materials science to characterize the microscale. However, these methods compromise the volume of interest to achieve a higher resolution. Dark-field (DF) contrast imaging is being investigated to characterize building materials in length scales smaller than the resolution of the imaging system, allowing a direct comparison of features in the nano-scale range and overcoming the scale limitations of the established characterization methods. This work extends the implementation of a dual-phase X-ray grating interferometer (DP-XGI) for DF imaging in a lab-based setup. The interferometer was developed to operate at two different design energies of 22.0 keV and 40.8 keV and was designed to characterize nanoscale-size features in millimeter-sized material samples. The good performance of the interferometer in the low energy range (LER) is demonstrated by the DF retrieval of natural wood samples. In addition, a high energy range (HER) configuration is proposed, resulting in higher mean visibility and good sensitivity over a wider range of correlation lengths in the nanoscale range. Its potential for the characterization of mineral building materials is illustrated by the DF imaging of a Ketton limestone. Additionally, the capability of the DP-XGI to differentiate features in the nanoscale range is proven with the dark-field of Silica nanoparticles at different correlation lengths of calibrated sizes of 106 nm, 261 nm, and 507 nm.

## Introduction

Cutting-edge research in building material science aims to improve the durability of existing materials and develop new materials, pursuing efficiency and sustainability. In that attempt, the use of effective techniques to characterize the internal structure at different length scales is key. Looking at the fine features that compose the internal structure of natural building materials, such as stones and wood, gives us the means to understand their performance and durability. In that attempt, several techniques are used to analyze the structure of the materials.

Quantitative techniques such as Mercury injection capillary porosimetry (MICP)^1–3^ are used to characterize the structure of mineral materials. The precision of the reported pore throat is expressed with an uncertainty of tens of nanometers by injecting mercury into the pore structure under increasing pressure. However, this technique is destructive as the pore is damaged due to the induced pressure, the pore size is underestimated as closed pores are not studied, and no morphological information can be retrieved.

Imaging-based characterization techniques have gained more attention as effective tools to describe the morphology, composition, and structure heterogeneity of building materials. Each imaging technique has its advantages and limitations. For instance, scanning electron microscopy (SEM) allows a topographic description of the surface of a sample with resolution up to tens of nanometers^4,5^. A 3D visualization is built up with focus ion beam nanotomography (FIB-nt), which creates a 3D image of the sample from several cross-sections of SEM images, sectioning the sample with a focused ion beam (FIB)^6^. Although high-resolution images are obtained by these techniques, the representative volume is limited to the microscale, they are destructive and they are time-consuming^7,8^.

Micro-computed tomography (μCT) and radiography are non-destructive X-ray imaging techniques that have gained acceptance for material characterization. Due to the penetration depth of X-rays, the internal structure of the material can be visualized and quantified at length scales of up to hundreds of nanometers in centimeter-scale volumes. In addition, complementary information about composition and structure is obtained by retrieving the phase signal. The drawback with μCT is the compromise of a representative macroscopic field of view (FOV) to achieve a higher spatial resolution. In building material characterization, i.e. to reach high sensitivity to features up to hundreds of nanometers the FOV goes down to the micrometer scale^9,10^.

Grating interferometry (GI) is a diffraction-based X-ray imaging technique that allows the simultaneous retrieval of the absorption, differential phase, and DF. It utilizes periodic phase modulation structures to produce an interference pattern downstream that is modified by the presence of a sample. Initially, Talbot grating interferometry (T-XGI)^11^ was developed in a synchrotron facility utilizing one phase grating to produce a diffraction pattern and an absorption grating closer to the detector to resolve the fringes. Later, Talbot–Lau interferometry (TL-XGI) permitted to translate the technique to a laboratory-based setup utilizing a source absorption grating to increase coherence^12^.

As visualization and quantification of the structure of materials at different length scales over a representative area of the sample is still a challenge, DF contrast for material characterization is investigated in an effort to improve the capabilities of X-ray imaging. The DF brings the advantage of non-destructively retrieving information of features below the finest spatial resolution of μCT systems, by obtaining scattering information of sub-resolution features of the sample while keeping a large FOV. Simultaneously, DF imaging adds complementary information to absorption and the phase imaging.

Previous studies using GI in synchrotron facilities have shown that DF is able to differentiate between two different nanoscale pore sizes^13^, and two materials in bricks with a sub-pixel resolution^10^. Different TL-XGI setups have been implemented and optimized for the phase contrast of biomaterials^14^. Nevertheless, the implementation of GI techniques in lab-based setups presents limitations for material characterization using DF imaging. The reduction in photon flux due to the implementation of the absorption grating and the variation of magnification of the sample when the sensitivity is tuned due to the fixed geometric arrangement of the gratings^15^ both hinder the possibilities of material characterizations.

In contrast, Dual-Phase X-ray Grating Interferometry (DP-XGI)^16^ is a technique that utilizes two phase gratings to generate a Moiré pattern downstream, improving dose efficiency as the fringes are directly resolvable by the detector. Furthermore, instead of moving the sample, the change of the inter-grating distance allows dark-field tunability while maintaining a constant magnification of the sample^17,18^.

In this work, the implementation of a lab-based DP-XGI setup aiming for a nanoscale characterization of building materials by tunable dark-field 2D imaging is presented. The imaging system has been optimized by a Fresnel-wave simulation framework^19,20^ for a low (LER) and a high energy range (HER) to investigate feature sizes in a range from tens to hundreds of nanometers in various types of materials.

## Methods

In this section, the image formation with a dual-phase interferometer is discussed and the definition of mean visibility reduction as a parameter of optimization is given. The dark-field contrast is explained based on its relationship with the unresolved micro-structure and with the protocol for its signal retrieval. Furthermore, we define the correlation length for the polychromatic system based on the geometrical parameters of the interferometer and the spectral properties of the system.

### Small angle scattering and dark-field contrast

The complex wavefront that describes the interaction of X-rays and matter under the projection approximation^21^ is defined as

1 $$\begin{aligned} U= U_{0} \exp \left( -ik \small \int _0^T n(x,y,z) dz \right) , \end{aligned}$$

where *U* is the resulting attenuated wavefront of an incoming wave $U_{0}$ after traveling through an object. Taking into account the thickness T and the complex index of refraction of the object defined as $n = 1- \delta + i \beta$, Equation  (1) becomes

2 $$\begin{aligned} U_{s}= U_{0} \exp (ikT) exp\left( -ik \small \int _0^T \delta (z) dz \right) \exp \left( -k \small \int _0^T \beta (z) dz \right) . \end{aligned}$$

In this expression, interactions within the material are approximated to a projection in the propagation direction *z*. Therefore, the linear integrals of $\beta (z)$ and $\delta (z)$ account for the absorption and the phase-shift of X-rays respectively. The wave number is $k= 2 \pi / \lambda$ with $\lambda$ the wavelength in vacuum.

The macroscopic variations of the wavefront due to the sample’s refractive index generate a measurable phase shift downstream. Small variations, on the other hand, account for the scattering of X-rays by unresolvable features with size *d*, in the regime where $\lambda /d \ll 1$. Lynch et al.^22^ proposed a redefinition of the refractive index to consider both the resolvable and non-resolvable interactions with the wavefront. Then, the refractive index is redefined as

3 $$\begin{aligned} n = n_{\textit{f}} + n_{\textit{s}}, \end{aligned}$$

where the sub-indices *f* and *s* correspond to the fine contribution of sub-resolution scatterers and the smooth contribution of the macrostructures, respectively. As a consequence, the wavefront in Eq. (2) is modified to account for the fine structure:

4 $$\begin{aligned} U =\exp (-i \Phi ) U_{s}, \end{aligned}$$

where $U_{s}$ is known as the contribution of the smooth features to the final wavefront, and $\Phi$, represents the phase-shift contribution of the fine structure of the material^22^.

GI techniques allow the retrieval of the absorption, the phase, and the scattering contrast generated by the fine features, i.e. the so-called dark-field image. These contrasts are obtained by a Fourier analysis of an interference pattern generated by the interaction of the wavefront with a periodic pattern and its modulation due to the presence of an object^12,16,23^.

In the following Section “Principles of dual-phase grating interferometry”, the fringe formation and the signal retrieval with DP-XGI are presented, enhancing the attributes this technique has among others for DF contrast in order to characterize building materials in a nanoscale range.

### Principles of dual-phase grating interferometry

**Figure 1:**
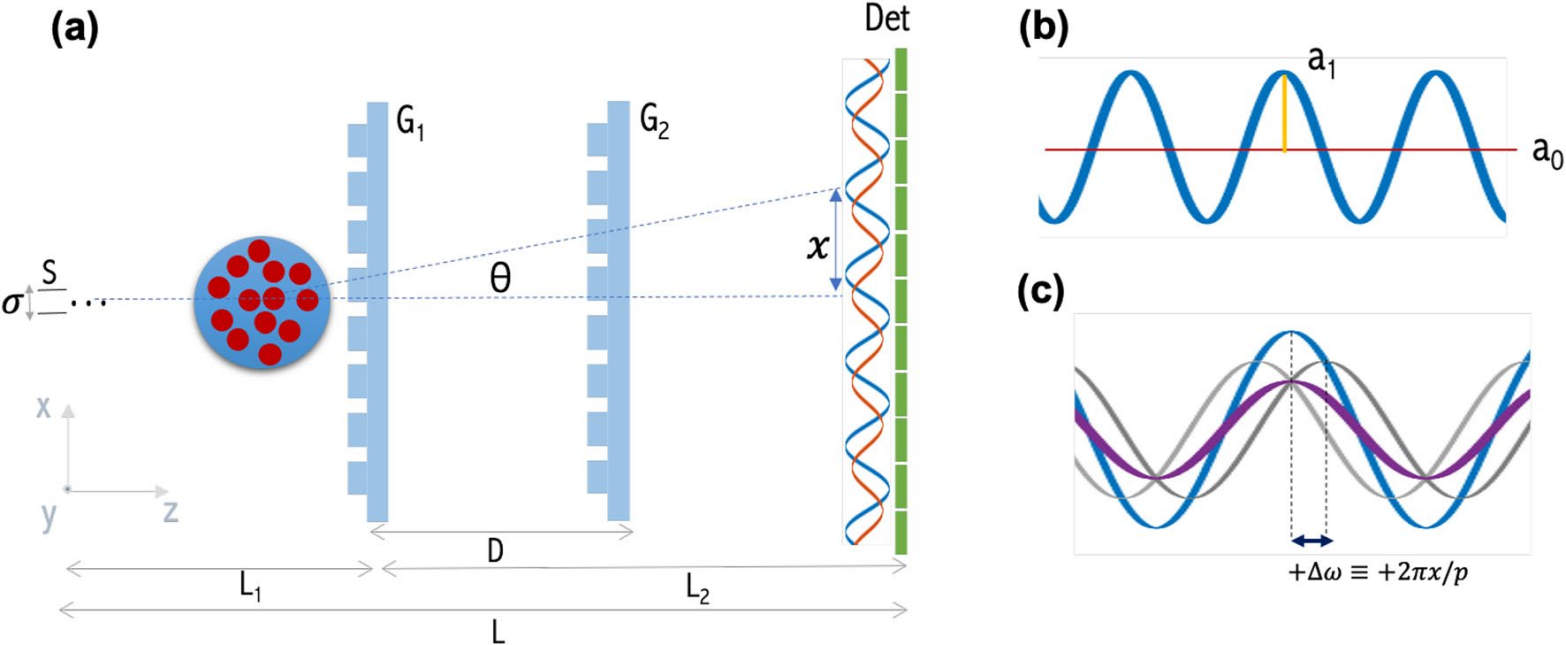
(**a**) DP-XGI setup geometry: $\text {L}_{1}$ is the distance between the source (S) and the first grating $\text {G}_{1}$, $\text {L}_{2}$ between $\text {G}_{1}$ and the detector (Det), and D is the distance between the two identical phase gratings $\text {G}_{1}$ and $\text {G}_{2}$. (**b**) is the reference interference pattern (blue line), where the mean visibility ($\bar{\text {V}}$) is defined by the ratio between $a_{1}$ and $a_{0}$. (**c**) Sub-pixel-sized features of a sample induce a phase shift $(\Delta \omega )$ of the modulation pattern, and the contribution of all random phases reduces the mean visibility of the final interference pattern (purple)^24^. The dark-field contrast (DFC) is obtained at different inter-grating distances D, as the ratio of the mean visibility with sample $\bar{\text {V}}_{s}$ (purple line), and without sample $\bar{\text {V}}_{f}$ (blue line).

A DP-XGI system^16^ is sketched in Fig. 1. It utilizes two phase gratings, $G_{1}$ and $G_{2}$, with the same period $\text {P}$ to generate an interference pattern downstream. The slight difference between the magnified wavefront that reaches the second grating and its period generates a Moiré pattern from which three complementary signals: the absorption, the differential phase, and the dark-field are retrieved. The fringe formation, the definition of effective energy, the correlation length in a polychromatic configuration, and the dark-field signal retrieval procedure are presented in this section.

#### Fringes formation and signal retrieval

DP-XGI is an imaging technique that can reach high sensitivity to features up to hundreds of nanometers in size with pixels in the order of tens of micrometers. This is achieved by utilizing two identical phase gratings with a period in the micrometer range, which generates a magnified interference pattern. The period of this interference pattern is on the order of hundreds of micrometers and resolvable by a detector downstream. A mathematical model for this fringe formation with a DP-XGI has been proposed by Yan et al.^25^ based on the Wigner distribution. According to this model, the interference pattern resolved by the detector is expressed by the Fourier coefficients of the transmission functions of each of the phase gratings.

Given the transmission functions of each phase-gratings as

5 $$\begin{aligned} \text {G}_{1}(x)=\sum _{s\ \in \ Z}a_{s}\exp \left[ i2 \pi \frac{sx}{p_{1}} \right] , \end{aligned}$$

and

6 $$\begin{aligned} \text {G}_{2}(x)=\sum _{r\ \in \ Z}a_{r}\exp \left[ i2 \pi \frac{rx}{p_{2}} \right] , \end{aligned}$$

where *s* and *r* represent the order of the Fourier coefficients for each of the gratings with Z an integer, and $p_{1}$ and $p_{2}$ are the period of each grating respectively.

When a wavefront interacts with the first grating, it is diffracted in s diffraction orders. According to the angular spectrum theorem, it can be interpreted as plane waves with amplitudes proportional to the Fourier coefficients $\text {a}_{\text {s}}$. After a propagation from $\text {G}_{1}$ to $\text {G}_{2}$, the wavefront is diffracted again by the second grating generating new tilted plane waves with amplitudes proportional to their Fourier coefficients $\text {a}_{r}$. At the detector plane, the different diffraction orders add up and produce interference patterns with different frequencies in combinations (*s*, *r*). As the pixel size of the detector $\text {P}_\text {det}$ works as a low pass filter, all the different $(\text {s},\text {r})$ diffraction patterns with higher frequency than $1/\text {P}_\text {det}$ are summed up as background. If a compact symmetric configuration is considered, where $\text {L}_{1}= \text {L}_{2}$ and the period of the gratings is the same and much smaller than the pixel size of the detector, only the diffracted orders $\text {s}=-\text {r}$ generate a magnified beating pattern resolvable by the detector^26^ with a first harmonic period of $\text {P}_\text {det}=\frac{ \text {PL}}{ \text {D}},$ where $\text {D}$ is the inter-grating distance, $\text {L}$ is the distance between the source and detector, and $\text {P}$ is the period of the phase gratings. Notice that the period is the same for both gratings.

As the DP-XGI works with a polychromatic X-ray tube, the spectral distribution of the source $\text {S}(\text {E})$ and the source size $\sigma$ influence the fringe formation modulating the mean visibility of the fringes ($\bar{\text {V}}$). The theoretical model for the interference pattern generated with a polychromatic illumination is defined as^25^

7 $$\begin{aligned} \text {I}\left( x, y; \text {E} \right) = \frac{ \text {I}_{in}}{ \text {M}_{ \text {G}_{1}}^{2}}\left[ 1+ \bar{\text {V}_{1}}\left( \text {E},\Delta \phi _{d}\right) \cdot cos\left( 2\pi \frac{x}{\text {P}_\text {det}} \right) + \bar{\text {V}_{2}}( \text {E},\Delta \phi _{d})\cdot cos\left( 4\pi \frac{x}{\text {P}_\text {det}} \right) \right] \end{aligned}$$

where the Fourier coefficients are considered up to the second order $\left( \bar{\text {V}_{1}}\, \text {and}\, \bar{\text {V}_{2}}\right)$. For clarity the bar stands for the mean value of the visibility over all the energy spectrum. Higher orders do not influence the fringes’ formation as the intensity pattern decreases at higher orders. To account for the polychromatic nature and the spot size of the source, the mean of each visibility coefficient $\bar{\text {V}_{1,2}}$ in Eq. 7 is photon energy dependant, and therefore spectrum averaged as

8 $$\begin{aligned} \bar{\text {V}}_{1,2}= \int \text {V}_{l}( \text {E},\Delta \phi _{d})\cdot \text {S}( \text {E})\text {d} \text {E}. \end{aligned}$$

Here $\text {S}(\text {E})$ is the effective spectrum weighted by the influence of propagation in air, interaction with the gratings, and the quantum efficiency response of the detector. $\Delta \phi _{\text {d}}$ corresponds to the phase modulation produced by the gratings to corresponding design energy of the system $\text {E}_{\text {{d}}}$ which is set as $\pi$ in this work, and $\text {V}_{l}( \text {E},\Delta \phi _{ \text {d}})$ stands for the visibility coefficients for specific photon energy.

#### Effective energy and correlation length

A DP-XGI is designed to generate a phase-shift $\Delta \phi _{d}$ to a wavefront with design energy $\text {E}_{ \text {d}}$. However, the polychromatic nature of the source implies that each energy of the spectrum will experience a different phase shift. Furthermore, the modulation pattern recorded downstream by a conventional integrating detector is the result of the superposition of all the different interference patterns generated by each energy and weighted by their visibility $\text {V}_{s}(\text {E},\Delta \phi _{d})$, as stated in Eq. 8.

The mean visibility is not homogeneous over the whole field of view^20^. For that reason, the effective energy $\text {E}_{\text {eff}}$ is both spatially dependent and spectrum dependent. For the calculation of the effective energy the source spectrum $S(\text {E})$, the filters in the setup, the scintillator response, the grating’s materials, and the visibility per energy $\text {V}(\lambda ,x)$ are required for its calculation as expressed in previous publications^19^. The values reported in the results were calculated with simulated values of the visibility spectrum^20^ and the target spectrum^27^.

#### Dark-field and tunability

As was disclosed in Section “Small angle scattering and dark-field contrast”, the dark-field signal originates from light scattered by the fine features of an object that cannot be resolved by an imaging system and introduce a random phase into the wavefront (Eq. 4)^28^. Strobl^24^ proposed a relationship between the scattering properties of the fine features given by the scattering vector $\text {q}=2 \pi x/\lambda \text {L}_{s}$, where *x* is defined by the scattering angle $\theta$ and the sample to detector distance $\text {L}_{s}$ according to Fig. 1 and the phase shift $\Delta \omega = 2 \pi x/\text {P}_{\text {det}}$ induced by the random phase $\phi$ to the interference pattern downstream in grating interferometry techniques. Here *x* is the shift of the interference pattern, with period $\text {P}_{\text {det}}$, due to a scattering angle $\theta$ according to Fig. 1a. Therefore,

9 $$\begin{aligned} \Delta \omega =\frac{\lambda \text {L}_{s}}{\text {P}_{\text {det}}}\text {q}= \xi \text {q}, \end{aligned}$$

where the correlation length $\xi =\frac{\lambda \text {L}_{s}}{\text {P}_{\text {det}}}$ is defined by the wavelength $\lambda$ of the incident X-rays, the distance of the sample to the detector $L_{s}$ and the period of the interference pattern at the detector plane $\text {P}_{\text {det}}$. For a configuration in which the sample is placed before $\text {G}_{1}$, the distance in Eq. 9 is redefined as $\text {L}'_{s}=(\text {L}_{1}+\text {L}_{2}-\text {L}_{s})\text {L}_{2}/\text {L}_{1}$^22,24^.

Considering the symmetric nature of the scattering function around $\text {q}=0$, a positive and a negative phase shift $\pm \, \Delta \omega$ of the interference pattern is generated, but cannot be resolved by the detector (see gray patterns in Fig. 1c). If one adds up the phase shifts from all the small features that scatter at all angles $\theta$, the interference pattern will exhibit reduced mean visibility (purple line in Fig. 1c). Dark-field is therefore measured as the reduction in mean visibility of the interference pattern downstream^16,24,29^.

In the following experiments, the mean visibility $\bar{\text {V}}$ is measured with the contribution of the first harmonic of the phase stepping curve, that is energy-weighted due to the polychromatic nature of the interferometer. Then, referring to Fig. 1b, the mean visibility estimated from experimental data is^16^:

10 $$\begin{aligned} \bar{V_{1}}=2\frac{a_{1}}{a_{0}}. \end{aligned}$$

The final DF is obtained from the ratio between the mean visibility modulated by the presence of the sample ($\bar{V}_{s}$) and the mean visibility of the modulation pattern without the sample $\bar{V}_{f}$^15,16^ defined as

11 $$\begin{aligned} DF = -\log \left( \bar{V}_{s}/\bar{V}_{f}\right) . \end{aligned}$$

The correlation length defined in Eq. (9) sets the sensitivity of the interferometer to different feature sizes. It is tuned by changing the wavelength, the period of the interference pattern, or the position of the sample. For a DP-XGI, the sensitivity is adjusted by changing the inter-grating distance D. Therefore, the period of the Moiré pattern $\text {P}_{\text {det}}$ changes while the magnification^16^ is kept constant. For a polychromatic illumination, an effective energy is defined as $\lambda _{\textit{eff}}= \frac{hc}{E_{\textit{eff}}}$.

A Real Space Correlation function RSCF of the random phases induced by the fine features within the object is obtained by measuring the dark-field at different inter-grating distances. The RSCF is related to structural information of the object^16,29,30^. At each inter-grating distance, i.e. each correlation length, the DF changes as the interferometer is sensitive to different scatterer properties. The shape of the scatterers modulates the behavior of the RSCF whereas the saturation point defines the larger fine feature that scatters^24^. Experiments with both neutrons and X-rays have been performed for the characterization of nanoparticles with GI in synchrotron facilities and lab setups. In these experiments, the RSCF was used to quantify the size of particles in hundreds of nanometers range^9,31,32^.

### DP-XGI implementation

**Figure 2:**
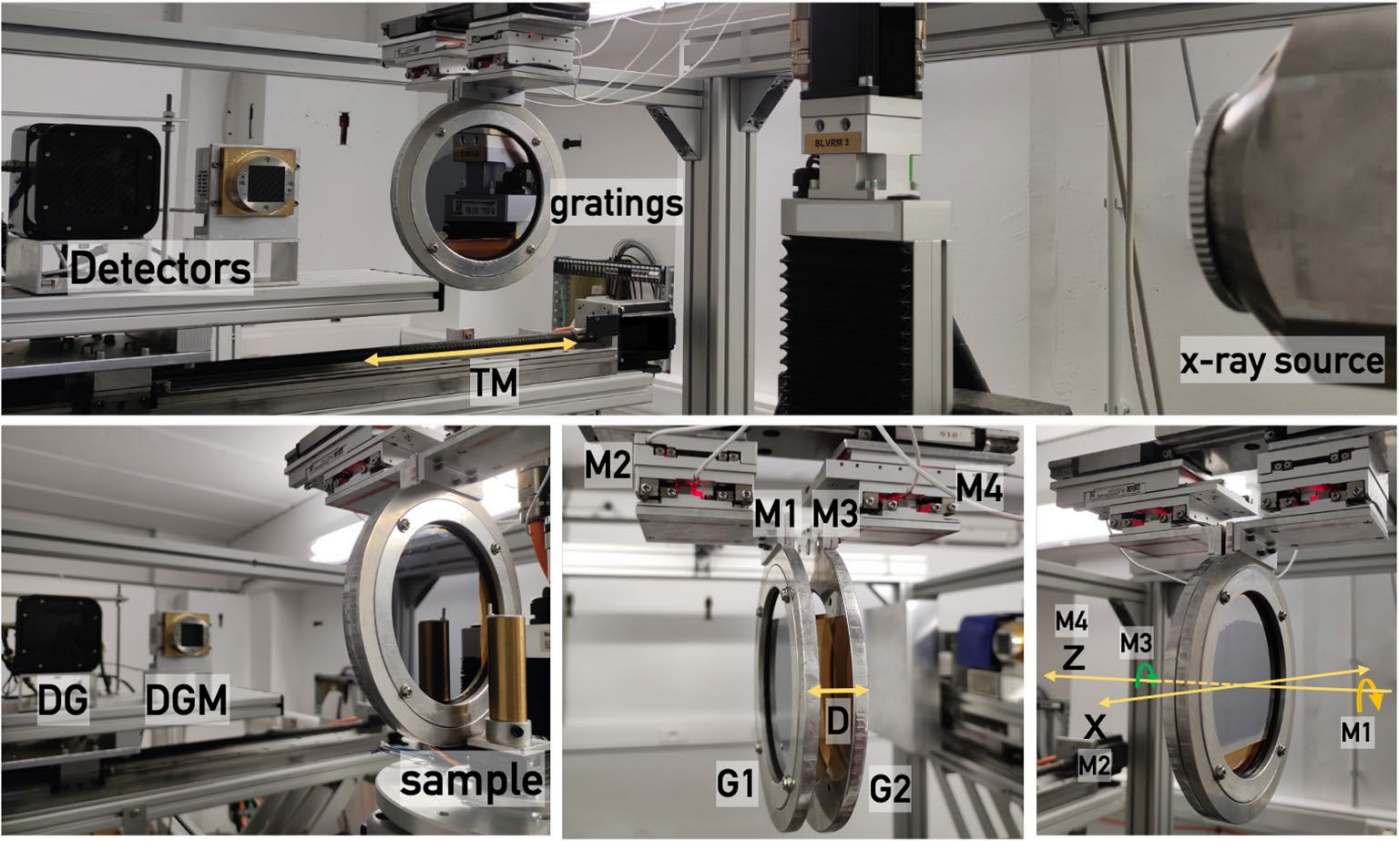
Configuration of the dual-phase interferometer. The two gratings, $\text {G}_{1}$ and $\text {G}_{2}$, are mounted over four different motors. $\text {M}_{1}$ and $\text {M}_{3}$ allow rotation around the z axis for alignment purposes, $G_{2}$ displacement in the z direction is performed with $\text {M}_{2}$ to change the inter-grating distance D, and $\text {M}_{1}$ moves $\text {G}_{1}$ in the *x* direction for phase stepping. Detection of the interference pattern can be performed by two different detectors (DG and DGM).

A dual-phase grating interferometer (DP-XGI) module is integrated in one of the X-ray micro-CT systems (Medusa) of Ghent University Centre for X-ray Tomography (UGCT, Belgium). As presented in Fig. 2, the micro-CT system is equipped with an X-ray source (FXE-160.51 tube, FEINFOCUS GmbH, Germany) with two heads: a high-power directional head and a transmission head^33^ allowing high-resolution imaging (0.9 μm), and two detectors, described in Section “Source and detector: conditions for optimal DF retrieval”, mounted on motorized linear stages that allow fast switching and highly accurate positioning between source and detector.

The interferometer module was designed to work in two different energy ranges and a Fresnel wave-front propagation simulation framework was carried out to select the optimal parameters^19^. For the low-energy (LER) configuration, the phase gratings are designed to produce a $\pi$ phase shift to a wavefront of 22.0 keV, while for the high-energy (HER) configuration, the phase gratings have a design energy of 40.8 keV and equal wavefront phase shift of $\pi$. Details on the fabrication are disclosed in Section “Gratings fabrication”. Considerations of the effective energy, the correlation length, and the dark-field retrieval are also included in that section.

#### Gratings fabrication

For each of the proposed configurations, a set of two gratings with a pitch of 1.0 μm was fabricated. The grating patterns were realized by Displacement Talbot lithography to ensure a uniform duty cycle over an area of $7\, \times 7~\text {cm}$ on a silicon substrate of $10~\text {cm}$ diameter^34,35^. Then, the high aspect ratio trenches were formed by deep reactive ion etching using the method developed in a previous work^36^. For a design energy of $22.0~\text {keV}$, the grating pattern was etched in silicon with a height of 28 μm and a duty cycle of 0.5 to produce a $\pi$ shift in the wavefront. Additionally, for the high energy range configuration, both gratings have trenches with a height of 9 μm filled by bottom-up gold electroplating to generate a $\pi$ shift at a design energy of $40.8~ \text {keV}$^37,38^. The gratings were characterized by scanning electron microscopy in cross-section in order to optimize the etching profile and the gold filling.

A superconformal process that yields bottom-up deposition in recessed features was used to Au fill the trenches in the gratings. The near-neutral pH, $\text {Na}_{3}\text {Au}(\text {SO}_{3})_{2}+\text {Na}_{2}\text {SO}_{3}$ electrolyte with its heavy metal p-block ion $\text {Bi}^{3+}$ additive for Au filling of high aspect ratio features has been substantially explored^37–44^. Void-free, bottom-up filling has been demonstrated in arrays of trenches as shallow as 3 μm to as deep as 305 μm with aspect ratios (depth/width) as low as 1.5 to greater than 60. The $\thickapprox 0.5$ μm wide trenches at 1.0 μm pitch filled for this study are narrower than the narrowest trenches, 0.65 μm wide at 1.3 μm pitch, filled previously^40,41^.

**Figure 3:**
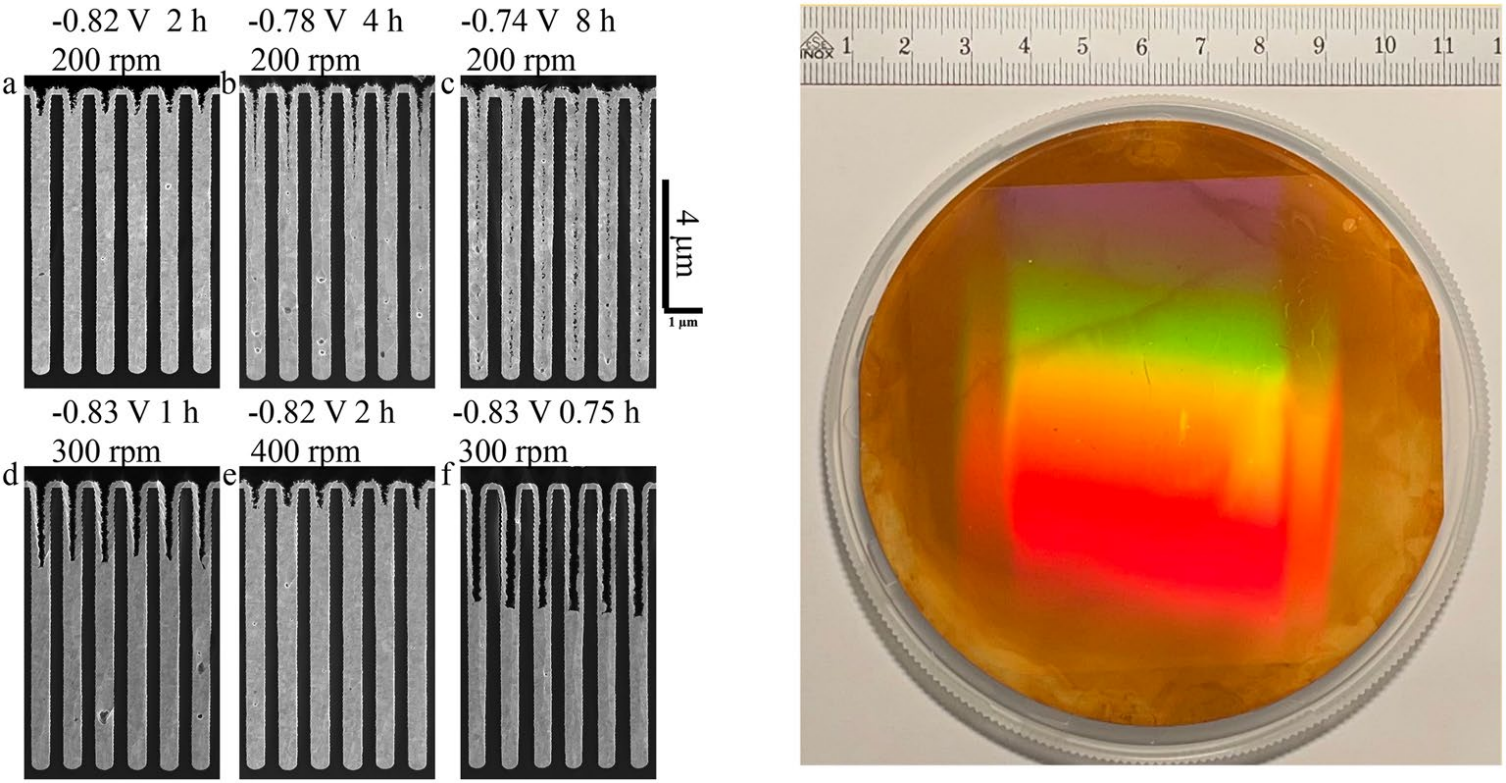
(**a**) SEM images in cross-section show the silicon template (dark) and the trench filling with gold (bright). The bottom-up filling was realized with Au electrolytes at pH 9.0, containing $0.64~\text {mol}\times \text {L}^{-1}~\text {Na}_{2}\text {SO}_{3}$ and $50~\mu \text {mol}\times \text {L}^{-1} \text {Bi}^{3+}$ for deposition at the indicated deposition potentials, times and substrate rotation rates. Gold concentrations were (**a**–**e**) $0.16~\text {mol} \times \text {L}^{-1}~\text {Na}_{3}\text {Au}(\text {SO}_{3})_{2}$ and (**f**) $0.08~\text {mol} \times \text {L}^{-1}~\text {Na}_{3}\text {Au}(\text {SO}_{3})_{2}$, (**g**) photo of one finalized full-sized grating filled with gold.

A survey of trench filling on fragments of the patterned wafer at varied electrodeposition conditions was conducted in a three-electrode electrochemical cell containing $40~\text {mL}$ of electrolyte; results for different deposition conditions are shown in Fig. 3. The electrolyte used in this work was $0.16~\text {mol} \times \text {L}^{-1}~\text {Na}_{3}\text {Au}(\text {SO}_{3})_{2} + 0.64~\text {mol}\times \text {L}^{-1}~\text {Na}_{2}\text {SO}_{3}$ of pH 9.0 containing $50~\mu \text {mol}\times \text {L}^{-1} \text {Bi}^{3+}$, although Fig. 3f demonstrates bottom-up Au filling with lower Au concentration. Filling of the complete (nominally) $7~\text {cm}\times 7~\text {cm}$ gratings used for the imaging study was accomplished in a cell containing $400~\text {mL}$ of electrolyte. All depositions were conducted at room temperature, with potentials measured relative to a $\text {Hg}/\text {Hg}_{2}\text {SO}_{4}/$ saturated $\text {K}_2 \text {SO}_{4}$ reference electrode separated from the main cell by a Vycor fritted bridge. Additional details are as in the earlier publications.

As in the previous studies, four distinctive characteristics of void-free filling in the $\text {Bi}^{3+}$ containing electrolyte were observed: (1) a potential-dependent “incubation period” of conformal deposition, (2) subsequent activation of deposition localized to the bottom surface of features, (3) continuing bottom-up deposition that yields void-free filling and (4) self-passivation of the active growth front at a distance from the feature opening. As it trended in previous studies with features of widely differing aspect ratio, deposition at more negative potential yields a transition from conformal filling with seam formation to the desired bottom-up superconformal filling^38–41^ (see Fig. 3a–c). A transition at even more negative potentials to increasingly large central voids^37–43^ is not obtained for the range of potentials examined here. Consistent with a recently published gradient-based model of the bottom-up Au filling^45^, the potentials of $-0.82~\text {V}$ and $-0.83~\text {V}$ yielding clear bottom-up Au filling in the relatively shallow and low aspect ratio trenches are $\thickapprox \text {100 mV}$ more negative than those for substantially deeper and higher aspect ratio trenches^37,38,43^. The narrow central seam that is especially evident after deposition at $-0.74~\text {V}$ reflects the conformal passive deposition that occurs absent bottom-up filling.

#### Motor stages

The two gratings were placed on top of motorized stages as shown in Fig. 2 to enable a precise translation of the gratings in different directions. The first grating $\text {G}_1$ is mounted on a piezoelectric goniometer (CGO-77.5, SmarAct GmbH, Germany.) with angular accuracy of 7 $\mu$-degree and 5 degrees  travel distance to allow tilting (M1). This goniometer is mounted on top of a piezo-drive linear stage (CLS-5252, SmarAct GmbH, Germany.) with $31~\text {mm}$ travel distance and $4~\text {nm}$ resolution (M2), which allows the displacement of $\text {G}_1$ in the direction perpendicular to the beam (axis *x* in Fig. 1) to perform the phase stepping acquisition explained in Section “Grating alignment”. Similarly, the $\text {G}_2$ grating is mounted on a second goniometer (M3) with the same characteristics as M1. Correspondingly, this goniometer is placed on top of another linear stage (CLS-5282, SmarAct GmbH, Germany.) to change the inter-grating distance (M4). This motor has a travel distance of $51~\text {mm}$ and $4~\text {nm}$ resolution. A sample stage is utilized to move the sample in and out of the beam automatically. To increase the sensitivity of the DF, this motor must keep the sample closer to $\text {G}_1$^17^.

#### Grating alignment

Grating alignment implies that, both gratings are simultaneously parallel to each other (zero relative angle between them) and aligned with the vertical coordinate axis of the detector along different inter-grating distances. To accomplish this, an automated alignment is performed using an algorithm developed in-house. This algorithm minimizes the angle between the gratings and ensures that they are not tilted. In addition, it calculates with an accuracy of millimeters the distance between the source and the first grating $\text {L}_{1}$ and with an accuracy of tens of micrometers the distance between the gratings D. The precision of these measurements allows an accurate calculation of the correlation length^46^.

#### Dark-field retrieval

The dark-field is retrieved by performing a phase-stepping procedure. A sinusoidal curve at each pixel is formed while stepping $\text {G}_{1}$ in the *x* direction according to Fig. 1a^11,47^. Then, by Fourier analysis of this stepping curve, the mean visibility is obtained. For the results presented here, interferograms are obtained at $\text {n}=~8$ phase steps to cover one period of the fringes, each with $15~\text {s}$ of exposure time. A customized module for the data acquisition framework has been developed to effectively operate within the existing LabVIEW$^{\circledR }$ software platform the CT scanner works with^48^.

#### Source and detector: conditions for optimal DF retrieval

To achieve a high mean visibility (Eq. 10) of the fringes in both configurations proposed, it is evaluated for different combinations of parameters (source and detectors). The source, a microfocus X-ray tube with a Tungsten target of $1~\mu$m thickness, is set in transmission mode. The versatility of this source allows it to work at different voltages in a range from $30~\text {kVp}$ to $120~\text {kVp}$.

Additionally, two detectors were used in the experiments: (1) an integrating detector (Photonic Science, 212045) with a Cesium Iodide CsI scintillator of 100 μm thickness and 16.4 μm pixel size in an array of $4096 \times 4096$, for a field of view (FOV) of $66.7 \times 66.7$ mm (2) an integrating detector (Photonic Science, 211024) with a GdOS scintillator of 6.6 $\text {mg/cm}^2$ thickness and a pixel size of 9 μm in an array of $4096 \times 4096$, for a FOV of $36.9 \times 36.9\, \text {mm}^2$. For clarity, in the following sections, the first detector is identified as DG and the second detector as DGM. Thus, experiments using high-energy gratings and the DGM detector are identified as HER-DGM and low-energy experiments using the DG detector as LER-DG.

The final performance of the DP-XGI compared to that predicted theoretically^19^ depends on factors including source size, detector blurring, and effective shape of the produced grating, which may deviate given the very challenging design parameters^20^.

## Results and discussion

### DP-XGI performance at low and high energy ranges

The performance of the DP-XGI is evaluated for different high-energy gratings-detector combinations HER-DG and HER-DGM and the low-energy combination LER-DG. The mean visibility $\bar{\text {V}}$, as defined in Eq. 10, is the figure of merit of the DP-XGI and it is evaluated for each case at different inter-grating distances, at every pixel of the field of view (FOV) and different voltages of the source: $70~\text {kVp}$, $80~\text {kVp}$, and $90~\text {kVp}$ for the HER configuration, and $30~\text {kVp}$, $40~\text {kVp}$, and $50~\text {kVp}$ for the LER.

**Figure 4:**
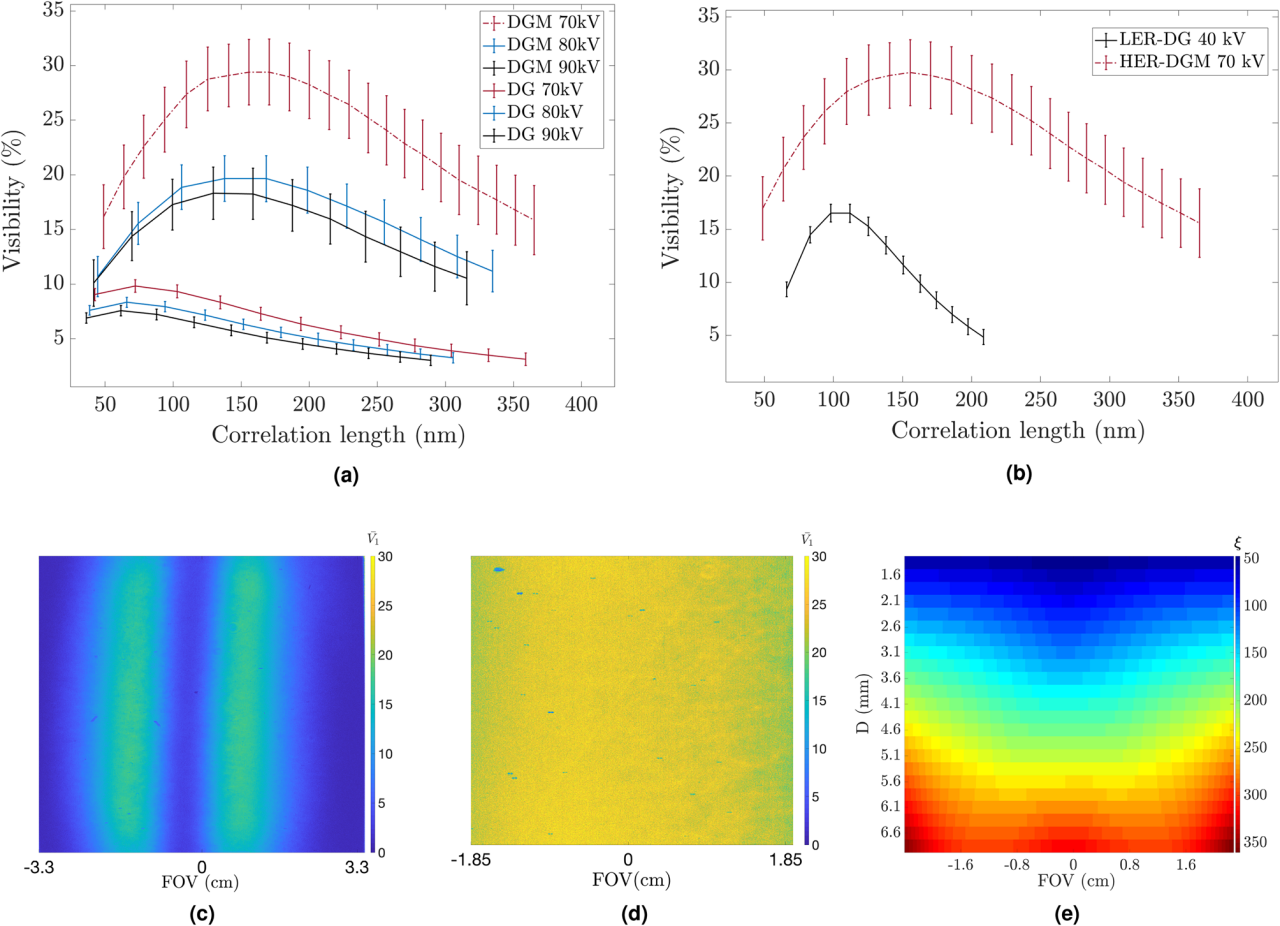
(**a**) Mean visibility at different correlation lengths for the HER configuration of the DP-XGI at different source voltages and two different detectors. Points are estimated as mean values of a chosen ROI; error bars are the corresponding standard deviation, (**b**) is a comparison of the LER and HER mean visibility. The spatial variation of the mean visibility is shown for LER in (**c**) and for HER-DGM in (**d**) at $\xi =93~\text {nm}$ and $\xi =150 ~\text {nm}$, respectively. The color map indicates the mean visibility ($\bar{V_1}$) obtained with Eq. 10. In (**e**), the color map indicates the correlation length at different FOV positions (x axis) vs different inter-grating distances (y axis).

Regarding the mean visibility maps, oscillations in the horizontal direction indicate that the mean visibility is spatially dependent on the opening angle of each energy due to the flat gratings and the polychromatic cone-beam nature of the DP-XGI^20^. Figure 4c shows a mean visibility map at a correlation length of 150 nm for the LER configuration and Fig. 4d for a HER configuration at a correlation length of 93 nm. Although the FOV for the latter configuration is smaller, the mean visibility distribution is more homogeneous providing a larger effective area. This behavior can be expected for all inter-grating distances.

The influence of the source voltage and the detector for the HER configuration is described in Fig. 4a. The mean visibility is higher along different correlation lengths for the HER-DGM configuration, despite the low energy response of the sensor, which utilizes a GdOS scintillator but has a smaller pixel size. Moreover, the highest mean visibility curve is given the tube set at $70~\text {kVp}$ as the design energy of the gratings matches the effective energy of this spectrum. In the case of the LER configuration, a similar analysis was performed, and the combination of $40~\text {kVp}$ with the DG detector (CsI scintillator and $16.4~\mu \text {m}$ pixel size) works best. Comparing LER-DG and HER-DGM, Fig. 4b shows how the HER-DGM configuration outperforms the LER-DG configuration while comparing mean visibility over a larger range of correlation lengths (from 40 nm to 380 nm). All the points of the RSCF curves reported in Fig. 4a and b are estimated as the average of a ROI where the range of mean visibility is high. For all cases, the mean visibility is plotted against the correlation length, and the uncertainty of the measurements is obtained as the standard deviation of the averages.

A second parameter that characterizes the interferometer is the correlation length. This geometrical parameter defines the length scale to which the DP-XGI is sensitive, i.e., the size of the fine features the dark field can differentiate. It is inversely proportional to the period of the modulation pattern $\text {P}_{\text {det}}$ and the energy of the imaging system (Eq. 9). In a DP-XGI, the polychromatic and cone-beam illumination nature of the imaging system implies that the effective energy, and therefore the correlation length, are energy spectrum and spatial dependant. Hence, the effective energy is measured as the average of each photon energy of the spectrum weighted by its quantum efficiency and the mean visibility spectrum $\textit{S}(\lambda$), as it is defined in a previous work^19^. The spectrum parameters were obtained with a Monte Carlo simulation reported by Dhaene^27^, and the mean visibility was obtained with the wave propagation cone illumination framework reported by Tang^20^. As an example, Fig. 4e shows how the correlation length for the LER configuration is spatially dependent as it changes horizontally for different points of the FOV and vertically for different inter-grating distances. The same procedure is followed to estimate the HER correlation length map which presents analogous behavior. The correlation length maps obtained for each configuration were averaged at each inter-grating distance and ROI to obtain the values reported in each of the plots of Fig. 4.

Additionally, the sensitivity of the interferometer to detect a mean visibility reduction of the fringes is measured as defined by Spindler et al.^49^, as $\tau =\text {V}_{0}\sqrt{\text {nI}_{0}}$. Here, $\text {n}$ is the number of phase steps, $\text {I}_{0}$ is the zero order of the Fourier coefficients of the phase-stepping curve, which correspond to the number of counts, and $\text {V}_{0}$ is the mean visibility^49^. This magnitude accounts for the influence of the quantum efficiency of the scintillators as it is proportional to the number of counts detected. For the DP-XGI configurations reported, the mean sensitivity over different correlation lengths is obtained. The LER configuration has a mean sensitivity of $66.1\pm 21.7$, closer to that of the HER-DG estimated as $67.4\pm 5.4$. Although the mean visibility is higher for the HER-DGM, its sensitivity is lower than the other two configurations with $49.3\pm 7.7$ on average. These results are to be expected since the GdOS scintillator is more efficient at lower energies and therefore less sensitive to energies at which the HER configuration operates. Nevertheless, one can increase the exposure time with HER-DGM to increase the sensitivity as flux is not a limiting factor in material characterization. However, the mean visibility of HER-DG is fixed for every configuration and depends on geometrical parameters. Therefore, in the context of this work, mean visibility is a crucial discriminating factor when choosing the optimal configuration.

Finally, sample parameters are key factors for optimal dark-field retrieval in each of the configurations. The thickness and the absorption coefficient define the correlation length range in which the dark-field contrast is not saturated^49^. Therefore, the samples used in the experiments in the following sections were chosen to avoid saturation at correlation lengths where the interferometer is more sensitive. In Section “Natural wood”, it is shown that a LER-DG configuration is suitable for characterizing materials with low absorption coefficients. On the other hand, the measurements on a limestone presented in Section “Ketton limestone” underline the good performance of the HER configuration for the characterization of high-denser materials.

Table 1 summarizes the setup parameters used for each configuration. The DP-XGI system reported in this work is unique for its versatility and ability to operate in two energy ranges allowing the characterization of materials with different electron densities. In addition, with the correlation length it achieves, features can be studied at length scales from tens to hundreds of nanometers.

**Table 1:** Different component combinations of the DP-XGI implemented to work in low and high energy modes for imaging system characterization. The interferometer is set with a 1 m source-detector distance and a symmetric configuration.

	Energy range		Gratings			Detector	System parameters		
	Source	${E}_{{D}}$ (keV)	Pitch (μm)	Material	Scintillator (μm)	Px size (μm)	Sensitivity (a.u.)	Max visibility (%)	$\xi$ range (nm)
LER-DG	W 40 kVp	$22 \pm 1$	1.0	Silicon (Si)	CsI - 22	16.4	$66.1\pm 21.7$	$\sim 16$	66–208
HER-DG	W 70 kVp	$40.8 \pm 2.1$	1.0	Gold (Au)	CsI - 22	16.4	$67.4\pm 5.4$	$\sim 10$	48–359
HER-DGM	W 70 kVp	$40.8 \pm 2.1$	1.0	Gold (Au)	GdOS - 8.3	9	$49.3\pm 7.7$	$\sim 30$	66–365

### Quantification of DFS

Monodisperse Silica ($\text {SiO}_{2}$) spheres with diameters of 166 nm, 261 nm, and 507 nm (Cospheric LLC, USA) were used to evaluate the capability of the DP-XGI to differentiate features in the nanoscale range. Experiments were performed with the LER configuration, and a beam hardening correction was implemented based on the algorithm developed by Tang^50^ considering a density of $2.0 \ \text {g/cc}$ for the spheres reported by the manufacturer.

**Figure 5:**
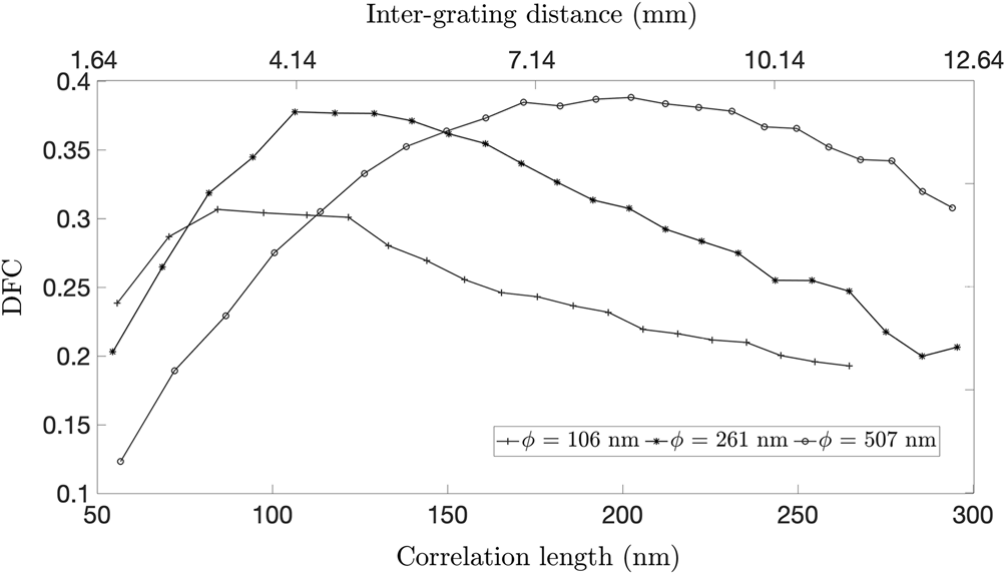
Real space correlation function of Silica nanoparticles of 166 nm, 261 nm, and 507 nm retrieved with the LER configuration of the DP-XGI and beam hardening corrected.

Figure 5 shows how the real space correlation function RSCF is different for each of the nanoparticles and the maximum DF is reached at a different correlation length. This behavior is expected as the correlation length in which the contrast saturates corresponds to the size of the largest fine feature, as was pointed out in Section “Dark-field and tunability”. These results are in good agreement with the theoretical model proposed by Yashiro^29^ and Lynch^22^, where structural information of the cluster of spheres can be obtained from the RSCF. Therefore, the results obtained with the DP-XGI prove that this interferometer can differentiate features in a nanoscale range. The plots in Fig. 5 also suggest that for the particular case of the DP-XGI, the DF decreases at higher correlation lengths after reaching the maximum. This is presumably caused by the loss of contrast at large distances due to the polychromaticity of the beam. Future work foresees the validation of the reported quantitative values with other imaging techniques to explain the particular behavior of the RSCF in DP-XGI. Therefore, the analysis of the DFC performed on building materials will be focused on qualitative differences.

### Material characterization with DF contrast

In this section, the performance of the interferometer is evaluated with the dark-field contrast retrieval at different correlation lengths, for wood in the LER configuration, and for a Ketton limestone in the HER configuration. A symmetric configuration of $1~\text {m}$ length was kept for all measurements. The X-ray tube was set at 40 kVp and 200 $\mu$A for the LER mode and at 70 kVp and 200 $\mu$A for the HER mode. For the phase stepping curve, interferograms of 15 s exposure time at 8 different $\text {G}_{2}$ positions were acquired, and 12 different inter-grating distances were evaluated.

#### Natural wood

**Figure 6:**
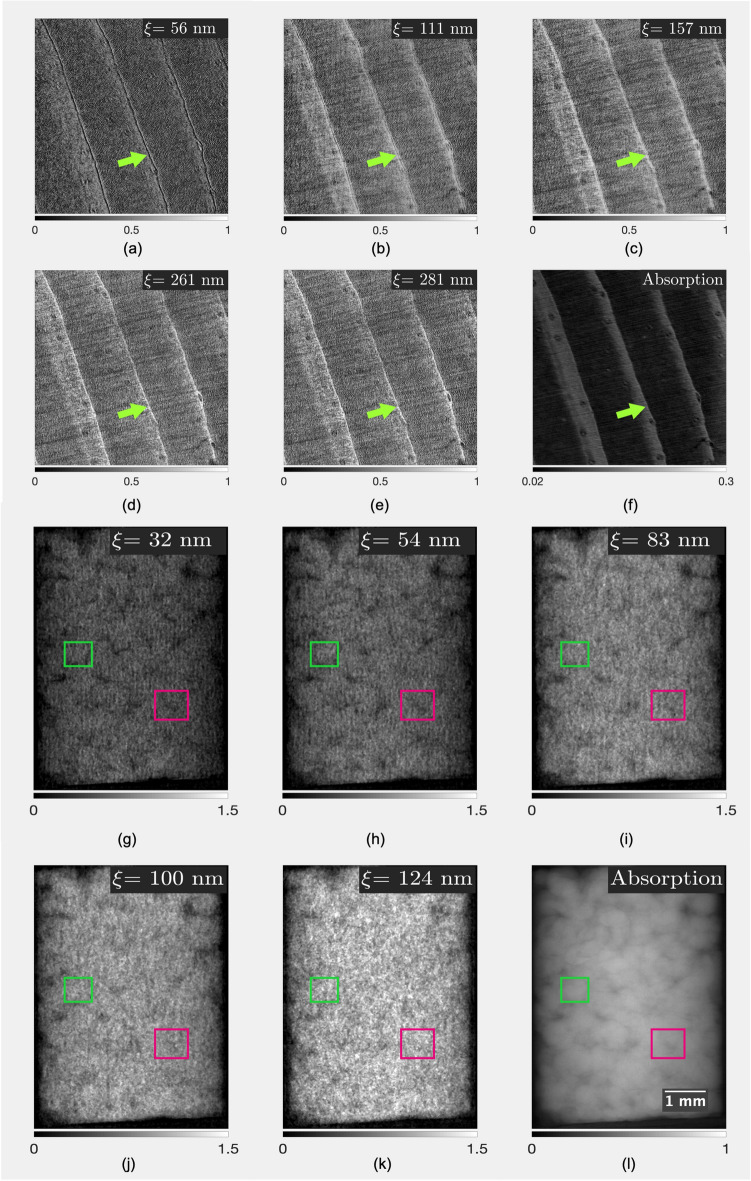
(**a**–**e**) DFC at different correlation lengths ($\xi$), and (**f**) the absorption contrast of a natural wood sample obtained with 40 kVp and two silicon gratings of 1.0 μm pitch and 28 μm height. (**g**–**k**) DFC and absorption (**l**) of a sample of Ketton limestone obtained at 70 kVp with gold-filled gratings of 1.0 μm pitch and 9 μm height. Arrows and small squares indicate some areas where the DF changes due to the presence of scatters in the nanoscale.

A natural wood sample (*Picea abies*) was cut out from a commercially available wood plank, for the examination of which no regulations apply. The sample dimensions are $(10\times 10\times 1.2)~\text {mm}^{3}$ with the short dimension taken in the tree’s radial direction, to have the elongated cells laying upright in the sample. The sample was positioned with several growth rings within the field of view, thereby capturing variations with early as well as late seasonal growth variations. In addition to the growth rings visible in the attenuation signal, Fig. 6a–f shows how the DF changes at different correlation lengths. Note particularly that at the edge of a growth ring (which displays a change in density), the DF contrast increases with the correlation length. This suggests that DP-XGI is sensitive to the nanoscale structures of the natural cell walls. The results are in good agreement with previous studies performed with X-ray small-angle scattering (SAXS), where growth year changes in a nanoscale range were reported^51,52^. As such, DP-XGI opens the door to non-destructive studies of phenomena such as heating, compression, and watering of wood in a nanoscale range, pursuing the translation of these experiments performed in synchrotrons to table-top setups.

#### Ketton limestone

The performance of the HER configuration is exhibited with the retrieval of the DF of a Ketton limestone of 3 mm diameter measured with the HER-DG configuration. Figure 6g–l shows local changes of the DF contrast with increase of the correlation length and its saturation around a hundred nanometers correlation length, suggesting the presence of scatterers in that scale range. SEM images of the same sample were obtained (see Figure S1 on the Supplementary material to verify the presence of scatters in the nanoscale. The images show clusters of grains hundreds of micrometers in size having internal features 10- to 100-fold smaller. The comparison of the Ketton limestone with the two techniques validates the capability of the DP-XGI with the HER configuration to identify the presence of nanoscale features. These qualitative results open the doors for further research in mineral material characterization in the nanoscale range. For example, adapting the method proposed by Blykers^13^ for the classification of pore sizes utilizing a lab-based setup instead of a synchrotron facility to differentiate between two different pore sizes comparing two different RSCF.

## Conclusion

The design parameters and implementation protocol of a dual-phase X-ray interferometer that allows dark-field imaging sensitive to features in a nanoscale range were presented. Correlation lengths of hundreds of nanometers can be reached, allowing the characterization of features with a size below the spatial resolution of the imaging system. Three different configurations have been implemented to characterize mineral and wood materials in the nanoscale with samples of tens of millimeters in size. Additionally, the robustness of the imaging system to differentiate features on the nanoscale is demonstrated using the real space correlation function of Silica particles with diameters of hundreds of nanometers.

The good performance of the DP-XGI for building materials characterization in a nanoscale range, with samples of millimeter range size, is validated with dark-field images at different correlation lengths of pine wood for the low energy range (LER) and Ketton limestone for the high energy range (HER). The results show that the reported configurations can provide qualitatively structural information in the nanoscale range. Moreover, the implementation of high-energy gratings allows to study features in a larger range of correlation lengths and highly dense materials (with less penetration depth). These results show the feasibility of material characterization in the nanoscale with the dark-field contrast retrieval in a lab-based setup with a dual-phase interferometer, and pave the way for quantitative studies of their internal structure, which is the focus of following works.

### Supplementary Information

Supplementary Information.

## Data Availability

The datasets acquired and analyzed during the current study are available from the corresponding author upon reasonable request.
