# Supplementary material for: Implementation of a dual-phase grating interferometer for multi-scale characterization of building materials by tunable dark-field imaging
Source: Sci Rep. 2024 Jan 3;14:384. doi: 10.1038/s41598-023-50424-6 (PMC10764912; doi:10.1038/s41598-023-50424-6)
Supplement: Supplementary file 1 — Supplementary Information. [file 41598_2023_50424_MOESM1_ESM.pdf]

Implementation of a dual-phase grating interferometer for multi-scale characterization of building materials by tunable dark-field imaging

Caori Organista, Ruizhi Tang, Zhitian Shi, Konstantins Jefimovs, Daniel Josell, Lucia Romano, Simon Spindler, Pierre Kibleur, Benjamin Blykers, Marco Stampanoni, Matthieu N. Boone.

Supplementary material

Scanning electron microscope cross-sectional images (SEM) of the Ketton limestone sample used in the experiments with the HER configuration of the DP-XGI. The sample consists of grains of hundreds of micrometers in size clustered together creating a pore space also in the micrometer range. Porous areas, with less than a micrometer size, can be observed with a close-up of the internal part of the grain.

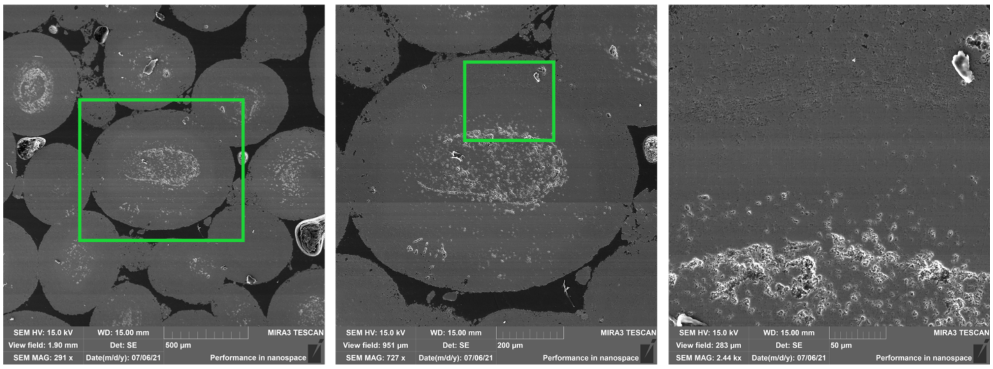

S 1: SEM images of a Ketton Limestone sample obtained at different magnifications: scale bars are 500 μm, 200 μm and 50 μm from left to right.
